# A H_2_S‐Generated Supramolecular Photosensitizer for Enhanced Photodynamic Antibacterial Infection and Relieving Inflammation

**DOI:** 10.1002/advs.202305183

**Published:** 2023-12-14

**Authors:** Jia Tian, Baoxuan Huang, Lei Xia, Yucheng Zhu, Weian Zhang

**Affiliations:** ^1^ Shanghai Key Laboratory of Functional Materials Chemistry East China University of Science and Technology Shanghai 200237 China

**Keywords:** anti‐inflammatory, hydrogen sulfide, photodynamic antibacterial, porphyrin, supramolecular photosensitizer

## Abstract

Photodynamic therapy (PDT) is a promising treatment against bacteria‐caused infections. By producing large amounts of reactive oxygen species (ROS), PDT can effectively eliminate pathogenic bacteria, without causing drug resistance. However, excessive ROS may also impose an oxidative stress on surrounding tissues, resulting in local inflammation. To avoid this major drawback and limit pro‐inflammation during PDT, this work prepared a supramolecular photosensitizer (**TPP‐CN/CP5**) based on host‐guest interactions between a cysteine‐responsive cyano‐tetraphenylporphyrin (**TPP‐CN**) and a water‐soluble carboxylatopillar[5]arene (**CP5**). **TPP‐CN/CP5** not only possesses excellent photodynamic antibacterial properties, but also shows good anti‐inflammatory and cell protection capabilities. Under 660 nm light irradiation, **TPP‐CN/CP5** could rapidly produce abundant ROS for sterilization. After the PDT process, the addition of cysteine (Cys) triggers the release of H_2_S from **TPP‐CN**. H_2_S then stops the induced inflammation by inhibiting the production of related inflammatory factors. Both in vitro and in vivo experiments show the excellent antibacterial effects and anti‐inflammatory abilities of **TPP‐CN/CP5**. These results will certainly promote the clinical application of PDT in the treatment of bacterial infectious diseases.

## Graphical abstract

A supramolecular porphyrin photosensitizer is prepared by host‐guest interactions, exhibiting excellent photodynamic antibacterial effect, but also good anti‐inflammatory and cell protection capability.

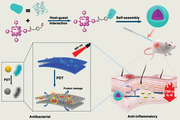

## 1 Introduction

The overuse of antibiotics has resulted in the development of antibiotic resistance in pathogenic bacteria, making the treatment of bacterial infectious diseases more challenging.^[^ ^1^ ^]^ In recent years, photodynamic antimicrobial therapy has garnered extensive attention for its non‐invasiveness, light‐controllable features, low systemic toxicity, and minimal drug resistance.^[^ ^2^ ^]^ Photosensitizers, which play a key role in photodynamic therapy (PDT),^[^ ^3^ ^]^ can transfer energy to surrounding oxygen molecules under the excitation of light at a specific wavelength, producing highly cytotoxic reactive oxygen species (ROS) that destroy biological molecules such as proteins and nucleic acids.^[^ ^4^ ^]^ The instantaneous production of large amounts of ROS achieves a potent antibacterial effect, reducing the risk of producing drug‐resistant bacteria.^[^ ^5^ ^]^ However, the excessive amount ROS acts as an exogenous stimulus, inducing oxidative stress in the surrounding tissues. This, in turn, triggers the release of a series of cytokines, including interleukin‐6 (IL‐6) and tumor necrosis factor‐alpha (TNF‐α), which causes local tissue inflammation, damaging proteins, nucleic acids, and lipids, and eventually resulting in cell death and tissue damage.^[^ ^6^ ^]^ Although the pro‐inflammatory side effects stemming from elevated ROS levels during PDT can seriously impede the development of clinical applications, they are often overlooked.^[^ ^7^ ^]^

Hydrogen sulfide (H_2_S) serves as a crucial endogenous gas transmitter, regulating physiological and pathological processes across the gastrointestinal, cardiovascular, nervous, and endocrine systems.^[^ ^8^ ^]^ Being an abundant physiological gaseous medium, H_2_S can effortlessly penetrate cell membranes without the need for specific carriers.^[^ ^9^ ^]^ Its roles and mechanism in cardiovascular protection, immune response, anti‐inflammatory, and anticancer activities have been closely investigated.^[^ ^10^ ^]^ Various platforms and advanced materials utilizing H_2_S donors have been designed for therapeutic and clinical purposes.^[^ ^8^, ^11^ ^]^ For example, Wan et al. introduced a zwitterion‐based H_2_S nanomotor designed to induce multiple acidosis in tumor cells, effectively inhibiting tumor growth.^[^ ^12^ ^]^ Lin and colleagues used dendritic mesoporous organosilica as a nanocarrier and a new kind of H_2_S gas generator, that they loaded with chloroperoxidase and sodium‐hyaluronate‐modified calcium peroxide nanoparticles to construct a tumor‐microenvironment‐responsive nanocomposite for H_2_S gas and trimodal‐enhanced enzyme dynamic therapy.^[^ ^13^ ^]^ In physiological environment, H_2_S suppresses the expression of inflammatory cytokines, including TNF‐α and IL‐6, by inhibiting crucial pro‐inflammatory transcription factors like phosphodiesterase and nuclear factor B (NF‐κB).^[^ ^14^ ^]^ Consequently, H_2_S can trigger endogenous antioxidant stress defense mechanisms, safeguarding cells against infection‐related oxidative stress.^[^ ^14a^ ^]^ Research and development of H_2_S‐based therapy is, thus, of great significance for treating inflammation‐related diseases.

**Scheme 1:**
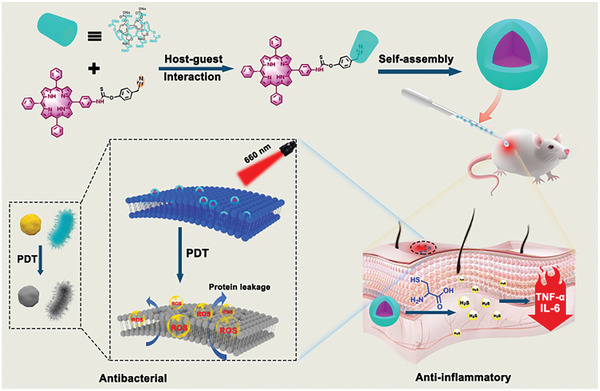
Schematic illustration of supramolecular porphyrin photosensitizer TPP‐CN/CP5 and its antibacterial and anti‐inflammatory activity.

In this study, we have developed a novel supramolecular porphyrin photosensitizer specifically triggered by cysteine (Cys) to release H_2_S, achieving the synergistic effect of photodynamic antibacterial and H_2_S anti‐inflammatory therapies. As illustrated in **Scheme** **1**, the photosensitizer consists of a cyano‐tetraphenyl porphyrin with a Cys‐responsive group (**TPP‐CN**) and the water‐soluble carboxylatopillar[5]arene (**CP5**). Functioning as a near‐infrared photosensitizer, **TPP‐CN** exhibits excellent ROS generation capability under 660 nm light excitation. Furthermore, the stability and biocompatibility of **TPP‐CN** could significantly be improved by forming the supramolecular porphyrin photosensitizer (**TPP‐CN/CP5**) through the host‐guest complex interaction between the cyano group and the water‐soluble macrocyclic host **CP5**. At the site of bacterial infection, the supramolecular photosensitizer **TPP‐CN/CP5** produces a substantial amount of ROS under light radiation, achieving the effect of photodynamic sterilization. Subsequently, in the presence of Cys, **TPP‐CN** releases H_2_S, which plays a role in resisting oxidative stress and relieving inflammation. Thus, **TPP‐CN/CP5** can simultaneously achieve bactericidal and anti‐inflammatory responses, facilitating the rapid recovery of the infected site. By intelligently combining antibacterial and anti‐inflammatory capacities, this strategy overcomes the pro‐inflammatory side effects of PDT, offering a novel prospect for PDT in the clinical treatment of infectious diseases.

## 2 Results and Discussion

### 2.1 Preparation and Characterization of Supramolecular Photosensitizer

**Figure 1:**
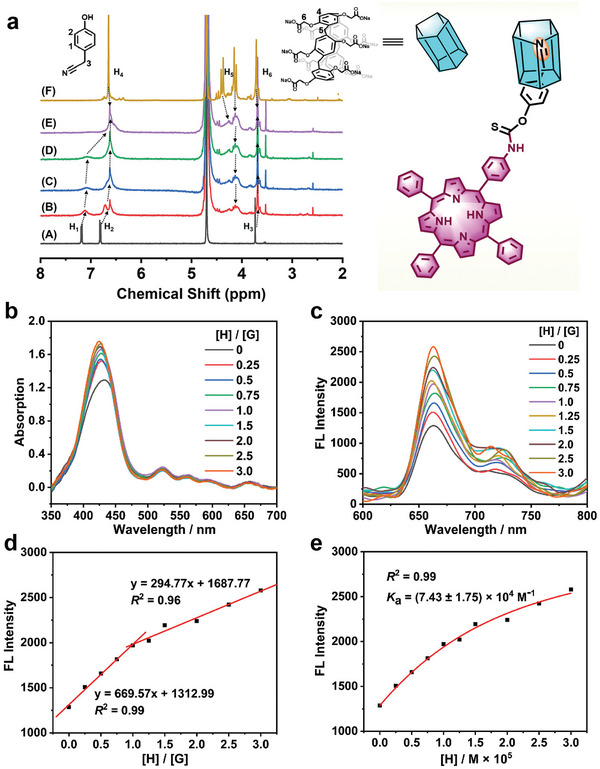
a) ^1^H NMR spectra of solutions of ***G_M_*** at a fixed concentration of 3.0 mm mixed with different concentrations of **CP5**: A) 0 mm, B) 1.0 mm, C) 2.0 mm, D) 3.0 mm, E) 5.0 mm, and F) ^1^H NMR spectrum of **CP5**. A schematic illustration of **CP5** (host) complexed with cyano group (guest) is shown on the right side of the ^1^H NMR spectra. b) UV–vis absorption spectra of **TPP‐CN** (1 × 10^−2^ mm) in aqueous solution in presence of different equivalents of **CP5**. c) Fluorescence spectra of **TPP‐CN** (1 × 10^−2^ mm) mixed with different equivalent of **CP5** (0‐3 equiv) in aqueous solution at room temperature. d) Job's plot for **CP5** and **TPP‐CN** in aqueous solution showed a 1:1 stoichiometry (according to fluorescence titration). e) Plot of fluorescence (FL) intensity as a function of concentration of **CP5**, where the red solid line was obtained from the non‐linear curve‐fitting.

The detailed synthetic procedures for the synthesis of cyano‐tetraphenyl porphyrin with a cysteine (Cys)‐responsive group (**TPP‐CN**) and carboxylatopillar[5]arene (**CP5**) are provided in Supporting Information (Scheme S1, Supporting Information). The host‐guest complexation of **CP5** and **TPP‐CN** was investigated through ^1^H NMR titration experiments. Owing to the poor solubility of **TPP‐CN** in D_2_O, the model guest ***G_M_*** (4‐hydroxyphenyl acetonitrile) was employed to form a complex with host **CP5**, as shown in **Figure** **1**a. Because of the shielding effect of the electron‐rich cavity of **CP5**, the signals related to the protons on ***G_M_*** (H_1_, H_2_, and H_3_) shifted upfield. Simultaneously, the phenyl protons (H_4_) and methylene protons (H_5_ and H_6_) on **CP5** slightly shifted upfield. These results confirmed that the host‐guest interaction between **CP5** and ***G_M_*** was robust enough to facilitate the versatile construction of supramolecular complexes.^[^ ^15^ ^]^

The formation of host‐guest complexation was further confirmed by UV–vis and fluorescence titration experiments. As the concentration of **CP5** increased, the absorption and fluorescence intensity of porphyrins gradually rose (Figure 1b,c), indicating that host‐guest complexation improved the dispersibility of **TPP‐CN** in aqueous solution. Simultaneously, the stoichiometry of complexation between **CP5** and **TPP‐CN** in aqueous solution was investigated using Job's plot method, which revealed a 1:1 stoichiometry between **CP5** and **TPP‐CN** (Figure 1d and Figure S6, Supporting Information). According to the non‐linear curve‐fitting, the association constant (*K*_a_) of **CP5** and **TPP‐CN** was calculated to be (7.43 ± 1.75) × 10^4^ m ^−1^ (Figure 1e), highlighting a strong binding affinity between **CP5** and cyano units. The excellent complexation of this host‐guest system was primarily attributed to hydrophilic‐hydrophobic interactions.

**Figure 2:**
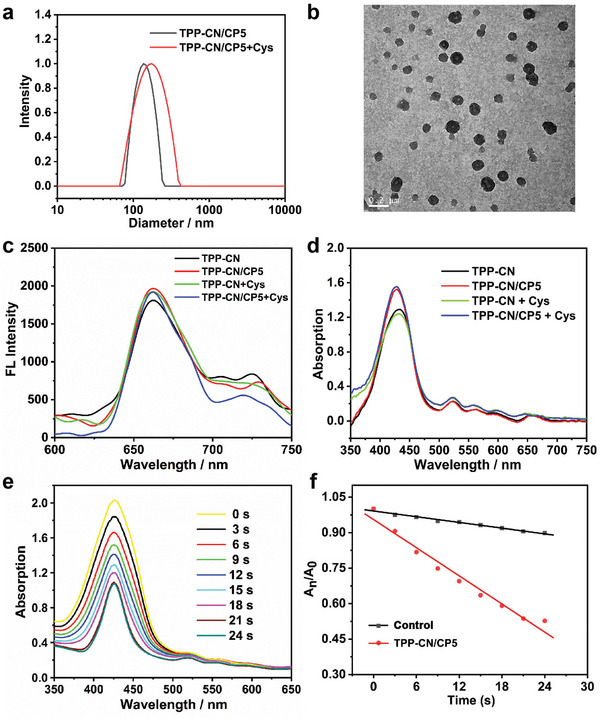
a) DLS results of **TPP‐CN/CP5** assemblies in aqueous solution with or without Cys. b) TEM image of **TPP‐CN/CP5** supramolecular nanoparticles. c) Fluorescence spectra of the **TPP‐CN**, **TPP‐CN/CP5** assemblies in aqueous solution at room temperature with or without Cys. d) UV–vis absorption spectra of **TPP‐CN** and **TPP‐CN/CP5** in aqueous solution with or without Cys. e) UV–vis spectra of 1,3‐diphenylisobenzofuran (DPBF) (detection of singlet oxygen production) in a **TPP‐CN/CP5** aqueous solution under 660 nm light irradiation. f) Percentage of degradation of DPBF in different solutions upon light irradiation.

Subsequently, supramolecular assemblies (**TPP‐CN/CP5**) were prepared by combining TPP‐CN and CP5 in aqueous solution via a precipitation strategy. The hydrodynamic size and morphology of **TPP‐CN/CP5** assemblies were characterized by dynamic laser scattering (DLS) and transmission electron microscopy (TEM). As shown in **Figure** **2**a,b, **TPP‐CN/CP5** assemblies exhibited a uniform spherical morphology with a hydrodynamic diameter of ≈140 nm. Upon incubation with Cys, both the size and polydispersity index of the assemblies slightly increased. Besides, the fluorescence spectra in Figure 2c and Figure S7, Supporting Information, revealed that the fluorescence intensity of **TPP‐CN/CP5** assemblies near 660 nm was stronger than that of **TPP‐CN**, which indicated that host **CP5** hindered the aggregation of porphyrins to some extent and enhanced the fluorescence of porphyrin molecules. The addition of Cys had minimal effect on the fluorescence properties of **TPP‐CN/CP5**. The UV–vis absorption spectra (Figure 2d) displayed the characteristic absorption of porphyrin, featuring a strong absorption peak near 425 nm (Soret band) and four weak absorption peaks between 500 and 670 nm (Q band).

Furthermore, the singlet oxygen (^1^O_2_) generation property of **TPP‐CN/CP5** under light irradiation was measured by using 1,3‐diphenylisobenzofuran (DPBF), a singlet oxygen scavenger. The absorbance of DPBF at 425 nm in a **TPP‐CN/CP5** solution gradually decreased with increasing irradiation time (Figure 2e). To evaluate the yield of ^1^O_2_ formation more quantitatively, we plotted the change in DPBF absorbance against irradiation time (Figure 2f). As shown in this plot, **TPP‐CN/CP5** possessed an outstanding ^1^O_2_ generation capacity, with a percentage of DPBF degradation reaching 48% within 30 s. It is also worth noting that the addition of Cys in a **TPP‐CN/CP5** solution significantly inhibited DPBF degradation under light irradiation. Interestingly, a similar observation could be made when we evaluated the change in DPBF absorbance of a **TPP‐CN/CP5** solution in presence of **H_2_S** (Figure S8, Supporting Information) for different irradiation time. We attributed this inhibition to the generation of H_2_S from the reaction between **TPP‐CN/CP5** and Cys, thereby quenching most of the ROS. To further confirm that this phenomenon was due to the antioxidant capacity of H_2_S rather than Cys, sodium tetrakis‐(4‐carboxyphenyl)‐porphyrin (**TCPP(Na_4_)**) was employed as a model photosensitizer to detect the degradation of DPBF with or without Cys (Figure S9, Supporting Information). The results showed that **TCPP(Na_4_)** demonstrated the same degradation ability for DPBF under light radiation regardless of the presence of Cys (Figure S10, Supporting Information). This indicated that the amount of ROS captured by DPBF was not affected by Cys, further supporting the conclusion that the low DPBF degradation capacity of **TPP‐CN/CP5** in the presence of Cys could be attributed to the release of H_2_S in response to Cys.

### 2.2 Cys‐Responsiveness and H_2_S Release from TPP‐CN/CP5 Assemblies

**Figure 3:**
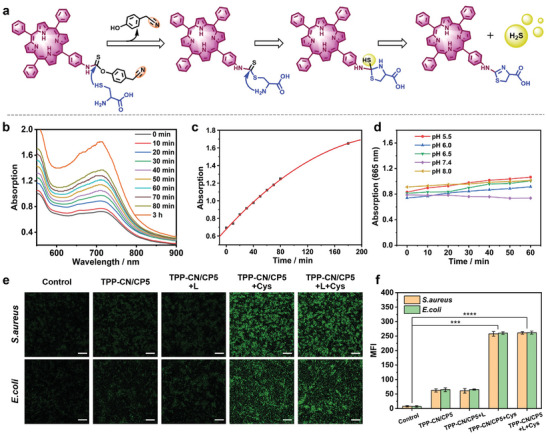
Evaluation of responsive release capacity of H_2_S. a) Proposed mechanism of H_2_S release from **TPP‐CN** triggered by Cys. b) UV–vis absorption spectra for detecting H_2_S release (MB method) at different incubation times. The concentration of **TPP‐CN** was 10 µg mL^−1^ and that of Cys was 100 µm. c) Plot of UV–vis absorption at 665 nm as a function of incubation time of **TPP‐CN** with Cys. d) Changes in UV–vis absorbance (at 665 nm) of H_2_S detection solutions (containing **TPP‐CN**) in the absence of Cys at different pH values at room temperature. e) Bacterial intracellular H_2_S detection using WSP‐1 as a probe after different treatments. Scale bar is 20 µm. f) Mean fluorescence intensities (MFI) of H_2_S probe fluorescence images. Results are expressed as mean ± SD (*n* = 3). One‐way ANOVA with a post‐hoc bonferroni's test, ***p ≤ 0.001; ****p ≤ 0.0001.

Because H_2_S is the third endogenous gas transmitter besides carbon monoxide and nitric oxide, its controlled release has attracted increasing attention.^[^ ^8a^ ^]^ The release of H_2_S can be triggered by specific stimuli such as thiols, light, enzymes, and ROS.^[^ ^16^ ^]^ In the present study, **TPP‐CN** reacted with Cys to release H_2_S, as depicted in **Figure** **3**a.

The colorimetric methylene blue (MB) assay was utilized to determine the Cys‐triggered H_2_S release of **TPP‐CN** and **TPP‐CN/CP5**. Figure 3b shows an obvious increase in UV–vis absorption as a function of incubation time of **TPP‐CN** with Cys, indicating an enhanced H_2_S release. The slow‐release process of H_2_S can further be observed in Figure 3c which shows the time‐dependent H_2_S release curve performed within 3 h. As mentioned in a previous study, the ideal H_2_S release process should be slow, continuous and controllable without disturbing the internal environment and homeostasis, or producing undesirable bioactive byproducts.^[^ ^17^ ^]^ **TPP‐CN/CP5** supramolecular assemblies presented a similar H_2_S release behavior triggered by Cys (Figure S11, Supporting Information). However, in the absence of Cys, no H_2_S was generated from **TPP‐CN** assemblies under neither acidic nor alkaline environments (Figure 3d and Figure S12, Supporting Information), indicating the good stability of **TPP‐CN** assemblies in different pH conditions. Similar behavior as a function of pH could also be observed for the supramolecular **TPP‐CN/CP5** assemblies in absence of Cys (Figure S13, Supporting Information). These results demonstrate that Cys plays an essential role in H_2_S production by **TPP‐CN** and **TPP‐CN/CP5** assemblies. For further investigation, we detected the responsive release of H_2_S from **TPP‐CN/CP5** in live bacterial cells using the H_2_S fluorescent probe Washington State Probe‐1 (WSP‐1).^[^ ^18^ ^]^ After pretreating bacteria with different solutions and WSP‐1, the channel images of the H_2_S probe were monitored by fluorescence microscopy (Figure 3e). As expected, almost no fluorescence was observed in the control group, and the bacteria exhibited weak fluorescence in the **TPP‐CN/CP5** groups due to the release of a small amount of H_2_S triggered by endogenous Cys. However, the green fluorescence became brighter, and the mean fluorescence intensity (MFI) strongly increased with addition of Cys (100 µm) (Figure 3e,f). These results validate that **TPP‐CN/CP5** can react with Cys for efficient H_2_S release.

### 2.3 In Vitro Antibacterial Activity and Biofilm Dissipation

**Figure 4:**
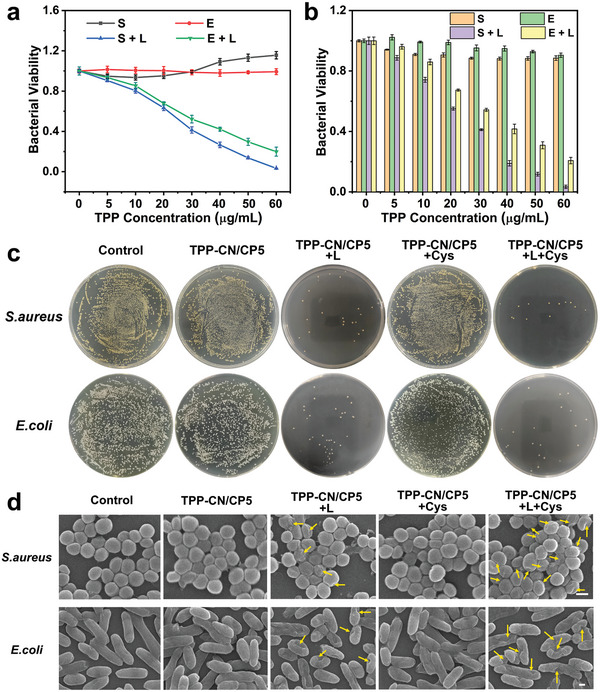
In vitro studies of antibacterial activity of **TPP‐CN/CP5**. a) In vitro toxicity of *S. aureus* (**S**) and *E. coli* (**E**) treated with **TPP‐CN/CP5** in the presence of Cys (100 µm). b) Bacterial viability of *S. aureus* (**S**) and *E. coli* (**E**) treated with **TPP‐CN/CP5. L** is short for light irradiation, representing that the sample was irradiated under light conditions. Values are mean ± standard deviation (SD) (*n* = 3). Student's *t*‐test was used for statistically significant analysis, **p ≤ 0.01; ***p ≤ 0.001. c) Plates images of *S. aureus* and *E. coli* colonies with different treatments. d) SEM images of *S. aureus* and *E. coli* with different treatments. Yellow arrows point to the obvious breakages of *S. aureus* and *E. coli*. Scale bar: 50 nm.

Inspired by the high ^1^O_2_ generation efficiency of **TPP‐CN/CP5**, the minimum inhibitory concentration (MIC) assay and the spread plate method were employed to evaluate the in vitro antibacterial activity against Gram‐positive bacteria (*S. aureus*) and Gram‐negative bacteria (*E. coli*). As depicted in **Figure** **4**a,b, **TPP‐CN/CP5** displayed a dose‐dependent antibacterial efficiency toward both *S. aureus* and *E. coli* under 660 nm light irradiation (600 mW cm^−2^, 15 min). Over 90% of *S. aureus* and 80% of *E. coli* were eliminated at a concentration of 60 µg mL^−1^ under light irradiation, while **TPP‐CN/CP5** displayed weak bacterial cytotoxicity in the absence of irradiation. Moreover, the addition of Cys after light irradiation had almost no effect on the photodynamic antibacterial performance of **TPP‐CN/CP5**. The disparity in survival rates between *S. aureus* and *E. coli* is mainly due to *E. coli* being Gram‐negative bacteria with an external cellular membrane linked by lipopolysaccharides, which reduces its sensitivity to reactive oxygen species.^[^ ^1c^ ^]^ Additionally, results from the spread plate method (Figure 4c) showed that the number of bacterial colonies was significantly low on the agar under 660 nm irradiation for both *S. aureus* and *E. coli*, showcasing the excellent PDT antibacterial ability of **TPP‐CN/CP5**.

**Figure 5:**
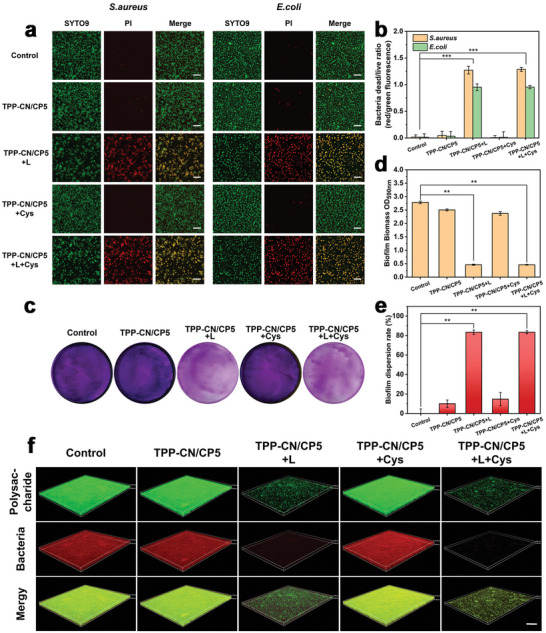
a) Live‐dead images of *S. aureus* and *E. coli* treated with PBS (control), **TPP‐CN/CP5** and **TPP‐CN/CP5+L** with or without Cys (100 µm). All bacterial cells were indicated by green fluorescence (SYTO9 channel), and the disruption of cell membranes was indicated by red fluorescence (PI channel). Scale bar: 10 µm. b) Quantitative analysis of red/green fluorescence signals, showing the dead/alive ratio of bacteria. Results are expressed as mean ± SD (*n* = 3), **p ≤ 0.01; ***p ≤ 0.001. c) The photographs of crystal violet (CV) stained MRSA biofilm in plates. d) OD_590 nm_ value of the remaining biofilm biomass after different treatment. e) Dispersion rate of biofilms with different treatments. Results are expressed as mean ± SD (*n* = 3), One‐way ANOVA with a post‐hoc bonferroni's test, **p ≤ 0.01; ***p ≤ 0.001. f) 3D CLSM images of biofilm treated with PBS (the control), **TPP‐CN/CP5** and **TPP‐CN/CP5+L** with or without Cys (100 µm). The polysaccharide on the biofilm and dead bacteria were stained with ConA‐FITC (green fluorescence) and PI (red fluorescence), respectively. Light irradiation: 660 nm laser light, 600 mW cm^−2^, 10 min. Scale bar: 20 µm.

To further visualize the destructive effect of PDT on bacteria, scanning electron microscopy (SEM) analysis was employed to observe the morphological changes of *S. aureus* and *E. coli* (Figure 4d). In both the control and non‐irradiated groups, the bacteria had regular body shapes with smooth surfaces and intact cell walls. In contrast, the cell membranes of bacteria treated with **TPP‐CN/CP5** and light were significantly wrinkled and collapsed, indicating the impaired or destroyed bacterial cellular integrity. Furthermore, the results of live‐dead staining analysis by confocal laser scanning microscope (CLSM) (**Figure** **5**a) confirmed that there was significantly more red fluorescence (staining dead bacterial cells) in light‐irradiated groups compared to the control and non‐irradiated groups, which primarily exhibited green fluorescence (staining live bacterial cells). Quantitative analysis of red‐green fluorescence intensity (Figure 5b) also showed that the number of bacterial deaths in the light‐irradiated groups were much larger than in the non‐irradiated groups. These findings were consistent with the above antibacterial results, emphasizing the remarkable PDT antibacterial efficiency of the **TPP‐CN/CP5** nanoparticles.

The formation of bacterial biofilm is a critical factor contributing to the development of bacterial resistance, which significantly hinders the recovery of infected site.^[^ ^19^ ^]^ Therefore, we further investigated the photodynamic biofilm dissipation capacity of **TPP‐CN/CP5**. The crystal violet (CV) staining method was employed to directly observe the effect of the supramolecular photosensitizers on biofilm dissipation (Figure 5c) and quantify the biofilm mass (Figure 5d). The stained biofilm plates and the residual biofilm biomass revealed that there was only a small amount of residual biofilm in the light groups (**TPP‐CN/CP5+L** and **TPP‐CN/CP5+L+Cys**), while the biofilm was relatively intact, with a certain thickness in all the non‐irradiated groups. Particularly, the dissipation efficiency of the biofilm treated by **TPP‐CN/CP5+L** and **TPP‐CN/CP5+L+Cys** both reached 84% (Figure 5e), demonstrating that ROS generated by **TPP‐CN/CP5** under light irradiation could effectively destroy the biofilm. The thickness and breakage of biofilms in different groups were observed by 3D images obtained by confocal microscopy (Figure 5f). Consistently, the biofilms treated with **TPP‐CN/CP5+L** and **TPP‐CN/CP5+L+Cys** exhibited fragmentation, and the bacterial density within the biofilm decreased dramatically. In contrast, the biofilms in other groups remained relatively dense and thick. The outstanding photodynamic biofilm dissipation capacity of **TPP‐CN/CP5** significantly contributed to its photodynamic antibacterial capability.

### 2.4 Anti‐inflammatory Activity of TPP‐CN/CP5

**Figure 6:**
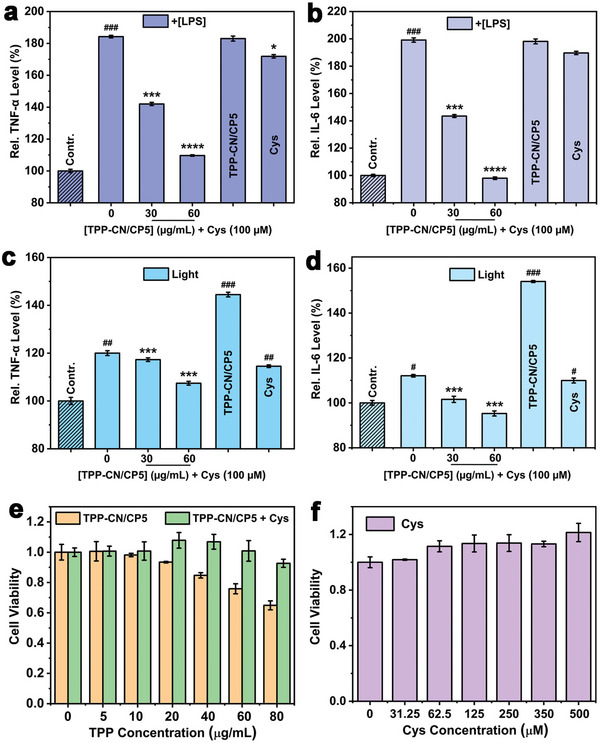
Expression of inflammatory cytokines a) TNF‐α and b) IL‐6 in LPS‐pretreated macrophage RAW264.7 cells. Results are expressed as mean ± SD (*n* = 3), ^###^ *p* ≤ 0.001 versus the control group; ***p ≤ 0.001 and ****p ≤ 0.0001 versus the 0 µg mL^−1^ of **TPP‐CN/CP5** group. The levels of c) TNF‐α and d) IL‐6 in light‐irradiated RAW264.7 cells (*n* = 3, mean ± SD). One‐way ANOVA with a post‐hoc Bonferroni's test, ^#^ *p* ≤ 0.05, ^##^ *p* ≤ 0.01, and ^###^ *p* ≤ 0.001 versus the control group; ***p ≤ 0.001 versus the **TPP‐CN/CP5** group. e) Cell viability of L929 treated with **TPP‐CN/CP5** and **TPP‐CN/CP5+Cys** (*n* = 3, mean ± SD). f) Cell viability of L929 treated with different concentrations of Cys (*n* = 3, mean ± SD).

Previous studies have reported that H_2_S can inhibit the production of inflammatory cytokines, achieving an anti‐inflammatory effect in the cell microenvironment.^[^ ^14a^ ^]^ We, thus, decided to take advantage of the excellent ability of **TPP‐CN/CP5** to release H_2_S in presence of Cys to alleviate inflammation induced by pro‐inflammatory cytokines and ROS. As a proof‐of‐concept, the anti‐inflammatory effect of **TPP‐CN/CP5** triggered by Cys in RAW264.7 cells was investigated. Lipopolysaccharide (LPS) was selected as the stimulus to establish the cellular inflammation model.^[^ ^6^ ^]^ The inflammatory response of RAW264.7 was evaluated through the expression levels of tumor necrosis factor‐α (TNF‐α) and chemokine interleukin‐6 (IL‐6) monitored by ELISA Kit. In comparison to the control group, the expression levels of TNF‐α and IL‐6 in RAW264.7 cells pretreated with LPS significantly increased. After incubating the pretreated cells with **TPP‐CN/CP5** and Cys, a dose‐dependent inhibition of TNF‐α and IL‐6 was observed. Moreover, the anti‐inflammatory effects of **TPP‐CN/CP5** without Cys and that of Cys alone were also evaluated; none of them attenuated the generation of TNF‐α and IL‐6 (**Figure** **6**a,b). These results indicate that **TPP‐CN/CP5** exhibited good anti‐inflammatory activity after being triggered by Cys, most likely due to the release of H_2_S.

Furthermore, solutions of **TPP‐CN/CP5**, Cys, and **TPP‐CN/CP5+Cys** with different concentrations of **TPP‐CN/CP5** upon 660 nm light irradiation were used to comparatively investigate the anti‐inflammatory protection capabilities against PDT‐aggravated ROS‐induced inflammation (Figure 6c,d). TNF‐α and IL‐6 were overexpressed after light irradiation in RAW264.7 cells incubated with **TPP‐CN/CP5** alone. In the absence of photosensitizers (the Cys group and the group of Cys 0 µg/mL + **TPP‐CN/CP5** 0 µm), the production of inflammatory factors induced by light irradiation was very limited. After the administration of **TPP‐CN/CP5+Cys**, the levels of TNF‐α and IL‐6 gradually decreased to reach those of the control groups, indicating an excellent anti‐inflammatory protection ability.

### 2.5 In Vivo MRSA‐Infected Therapy

Considering the desirable antibacterial properties and anti‐inflammatory effects mentioned earlier, the efficacy of the antibacterial and anti‐inflammatory performance of **TPP‐CN/CP5** was further evaluated in an MRSA‐infected mouse wound model. Before conducting in vivo antibacterial experiments, we first tested the biocompatibility of **TPP‐CN/CP5** and Cys by MTT cell assay. As shown in Figure 6e, the **TPP‐CN/CP5** assembly exhibited a certain level of toxicity to L929 cells as the concentration increased. However, the cell survival rate was significantly improved after the addition of Cys, possibly because the generation of H_2_S could promote anti‐apoptotic effects to protect the cells.^[^ ^17^ ^]^ The results in Figure 6e,f confirmed that the treatment of **TPP‐CN/CP5+Cys** was biocompatible and could be applied to subsequent in vivo studies.

**Figure 7:**
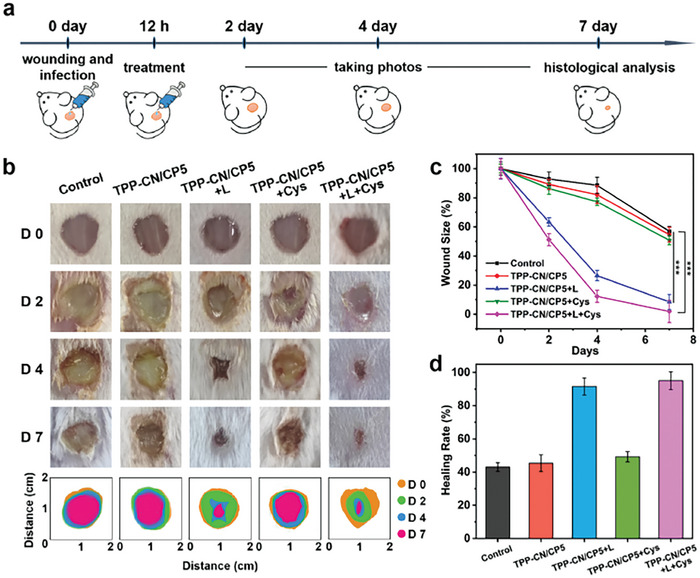
In vivo antibacterial efficacy study using mice wound model of MRSA infection. a) Schematic diagram of the in vivo antibacterial experiment. b) Photographs of MRSA infected wound sites on mice taken over the 7‐day post‐treatment experiments, for different treatments; Note: the scale bar for wound size is given at the bottom. c) Changes of infected wound sizes in mice of the different groups. d) Wound healing rate of mice in different groups. Values are mean ± standard deviation (SD) (*n* = 3), One‐way ANOVA with a post‐hoc Bonferroni's test,**p ≤ 0.01; ***p ≤ 0.001. (600 mW cm^−2^, 15 min).

The animal experiments were performed according to the protocol schematized in **Figure** **7**a. After wound modeling and bacterial infection, mice were randomly divided into five groups, which were given different treatments: phosphate buffered saline (PBS) (control group), **TPP‐CN/CP5** (dark group), **TPP‐CN/CP5+L** (light), **TPP‐CN/CP5+Cys** (dark group), and **TPP‐CN/CP5+L+Cys** (light). The evolution of the MRSA infection sites of mice in each group was monitored over a period of 7 days, and photographs of the wound sites are shown in Figure 7b. To better illustrate the variation in the wound healing effects of the different treatments, the corresponding wound sizes were measured. The wound healing trend could be observed through the change in wound size (Figure 7c and Figure S14, Supporting Information). In the group treated with **TPP‐CN/CP5+L+Cys**, there was no obvious suppuration in the wound, which gradually healed from day 0 to day 7, leaving a negligible small scar on the skin. However, the wounds of mice in other groups showed a certain degree of suppuration on the second day after MRSA infection. Additionally, suppuration in MRSA‐infected wounds for the control or dark groups became severe on day 4, and large wounds were still visible by day 7. Figure 7d illustrates that on the seventh day the infected wound healed by more than 90% after treatment with **TPP‐CN/CP5** under laser irradiation, especially in the **TPP‐CN/CP5+L+Cys** group, where the wound healing rate reached 98%, while the wound healing rate of other groups was relatively low. Wound healing rates of mice in each group on day 4 and day 7 were recorded (Figure S15, Supporting Information). Although **TPP‐CN/CP5+L** could also facilitate the wound healing process, the degree of wound recovery and relative wound size in mice showed that the bacterial inhibitory effect was enhanced when combined with Cys. This improvement was due to the release of hydrogen sulfide triggered by the addition of Cys to **TPP‐CN/CP5**, effectively inhibiting the expression of inflammatory cytokines in wound tissues after antibacterial PDT by **TPP‐CN/CP5**. Thus, the dual antibacterial and anti‐inflammatory effects jointly promoted wound healing.

**Figure 8:**
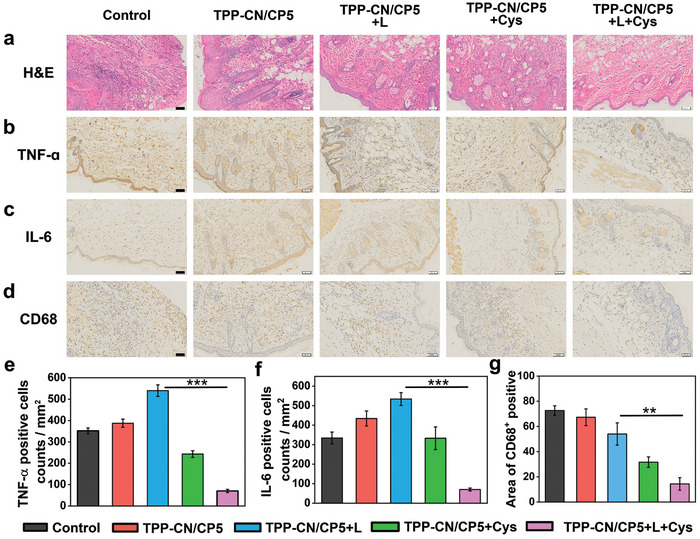
Histological and immunohistochemical evaluation of skin tissues after 7‐day treatments. a) H&E staining images of the infected skins harvested from different mice on seventh day post‐treatment. Immunohistochemical staining for b) TNF‐α and c) IL‐6 in the skin tissues of the infected areas. d) Immunostaining images of CD68^+^ expressed in the phagocytic macrophages. Scale bar: 50 µm. The corresponding quantification of positive cells with e) TNF‐α, f) IL‐6, g) CD68^+^. *n* = 3, One‐way ANOVA with a post‐hoc Bonferroni's test, **p < 0.01, ***p < 0.001.

Histological analyses using hematoxylin and eosin (H&E) staining were performed to evaluate the wound‐healing efficacy. As shown in **Figure** **8**a, the skin tissues of the control group and dark groups exhibited acute and extensive inflammatory states with a significant infiltration of inflammatory cells. Conversely, in the **TPP‐CN/CP5+L** group, there was a reduction in inflammatory cells, and the inflammatory foci became localized, which indicates that the inflammation was alleviated due to the elimination of pathogenic bacteria by PDT. The **TPP‐CN/CP5+L+Cys** group displayed fewer inflammatory cells and better‐formed fibers were observed, exhibiting healthy morphological features and suggesting excellent antibacterial and wound healing ability.

Subsequently, we further analyzed the expression of tumor necrosis factor‐α (TNF‐α) and interleukin‐6 (IL‐6), crucial inflammatory markers, in the wound tissues by immunohistochemical (IHC) evaluation. A trend similar to that observed with H&E staining can be seen in Figure 8b,c, where the relevant inflammatory factors were stained brown. The control group, dark groups, as well and **TPP‐CN/CP5+L** group, exhibited a wide range of TNF‐α and IL‐6 positive expression cells. In contrast, the expression of these pro‐inflammatory cytokines was significantly restrained in the **TPP‐CN/CP5+L+Cys** group. Similarly, consistent results were observed in skin slice images for each group by using CD68 as a reliable marker of macrophages with pro‐inflammatory activity (Figure 8d). Compared with the other four groups, the expression level of CD68^+^ in the **TPP‐CN/CP5+L+Cys** group was extremely limited, indicating that the mice from this group basically recovered to a healthy state without any obvious inflammatory reaction in the wound sites.

From the above experimental results, we can conclude that **TPP‐CN/CP5+L** could effectively eliminate local pathogens through PDT. However, the photodynamic antibacterial process can exacerbate inflammation, limiting the application of PDT in infectious diseases. PDT performed with **TPP‐CN/CP5+L+Cys**, on the other hand, decreases effectively the external stimuli of bacteria. Simultaneously, **TPP‐CN/CP5+L+Cys** alleviates the side effects of PDT by releasing H_2_S to inhibit the expression of pro‐inflammatory factors and regulate local inflammation.

## 3 Conclusion

In summary, a supramolecular porphyrin photosensitizer **TPP‐CN/CP5** with antibacterial and anti‐inflammatory functions was successfully designed and constructed, which solved the contradiction between ROS sterilization and pro‐inflammatory effect in PDT applications. **CP5** endowed the supramolecular **TPP‐CN/CP5** assemblies with good water solubility and biocompatibility, improving its ROS generation capability. In addition, **TPP‐CN** could be triggered by Cys to release H_2_S, which has excellent anti‐inflammatory ability. Therefore, **TPP‐CN/CP5+Cys** nanoplatform achieved the perfect combination of PDT sterilization and H_2_S anti‐inflammatory effects. The PDT antibacterial activity of **TPP‐CN/CP5** was confirmed by in vitro antibacterial experiments and MRSA‐infected wound experiments. Moreover, the results of the macrophage inflammation model and immunohistochemical evaluation showed that **TPP‐CN/CP5** significantly inhibited the expression of related inflammatory factors (TNF‐α and IL‐6) in the presence of Cys. The integration of PDT antibacterial and H_2_S anti‐inflammatory properties was effective, with excellent antibacterial activity, protecting normal cells from oxidative stress and inflammatory reactions. Thus, this antibacterial system sheds light on the future design and development of photodynamic antibacterial materials and provides a broad prospect for its future clinical application.

## 4 Experimental Section

### Self‐Assembly of TPP‐CN/CP5 in Aqueous Solution

**TPP‐CN** (1.0 mg, 0.001 mmol) was dissolved in 0.2 mL of DMF and then injected dropwise into a **CP5** aqueous solution (5 mL, 0.15 mm) under stirring. After stirring for 4 h, DMF was removed by dialysis (MWCO = 12 kDa) against deionized water (renewed fresh water four times). Finally, the concentration of the assembly was 0.36 mg mL^−1^.

### In Vitro Antibacterial Experiments

A bacteria suspension was diluted to 10^6^ CFU mL^−1^ with a sterile medium. Then a volume of 150 µL of the diluted bacterial solution was added into 96‐well plates and mixed with 50 µL of sample solutions with varying concentrations (dissolved in pure liquid medium). The irradiation duration and intensity were set as 660 nm laser light on the power of 600 mW cm^−2^ for 15 min (shortened as “+L” in group labeling). The 96‐well plates were incubated at 37 °C for 24 h after different treatments. A control group with only bacterial solution and medium was carried out. The group TPP‐CN/CP5 +Cys referred to the bacteria treated by both TPP‐CN/CP5 and Cys, but no light irradiation. After light irradiation, ʟ‐cysteine (100 µm) was immediately added to the TPP‐CN/CP5+L+Cys group. Subsequently, the bacteria suspension was diluted with PBS 1000 times and spread on the solid LB agar plate, followed by culturing at 37 °C for 12 h before colony forming units (CFU) counting and taking photos.

Triplicate analyses of each sample were performed and each experiment was carried out in duplicate.

### SEM Analysis

The bacterial suspension (10^9^ CFU mL^−1^) was washed with PBS, centrifuged (6500 rpm for 4 min), and dispersed in 1 mL of PBS. Next, the bacterial suspension alone or treated with **TPP‐CN/CP5** assembly (80 µg mL^−1^) was incubated in a shaking incubator (37 °C, 180 rpm). After 30 min, the latter was irradiated by 660 nm light for 15 min or not. After further incubation with Cys or not for 1 h, the bacteria were centrifuged to remove PBS and then fixed with 2.5% glutaraldehyde overnight. The glutaraldehyde was removed by centrifugation and the bacteria were washed with PBS three times. Subsequently, the obtained samples were dehydrated with ethanol solution in a graded series (30%, 50%, 70%, 80%, 90%, and 100%) for 15 min, respectively, and followed by being dispersed in ethanol. Ultimately, the specimens were dropped onto clean silicon slices and dried in the air before being observed by SEM.

### Live/Dead Bacterial Staining Assay

Bacteria were incubated in an LB medium overnight with shaking. Then 0.5 mL bacteria suspension was centrifuged to remove the medium and then dispersed in PBS containing **TPP‐CN/CP5** nanoparticles of 80 µg mL^−1^. The suspensions were irradiated by 660 nm light for 15 min or not after incubation for 1 h, and further treated with or without Cys (100 µm). The live/dead dye SYTO9 and PI were added in the dark and kept for 20 min. Finally, CLSM was used to observe the viability of bacteria.

### Statistical Analysis

One‐way ANOVA with a post‐hoc bonferroni's test was used for statistical analysis. The statistically significant difference between different groups was marked as ^∗^ *p*, ^∗∗^ *p*, and ^∗∗∗^ *p* for *p* < 0.01, 0.05, and 0.001, respectively.

## Conflict of Interest

The authors declare no conflict of interest.

## Supporting information

Supporting Information

## Data Availability

The data that support the findings of this study are available in the supplementary material of this article.
