# Supplementary material for: A H2S‐Generated Supramolecular Photosensitizer for Enhanced Photodynamic Antibacterial Infection and Relieving Inflammation
Source: Adv Sci (Weinh). 2023 Dec 14;11(9):2305183. doi: 10.1002/advs.202305183 (PMC10916657; doi:10.1002/advs.202305183)
Supplement: Supplementary file 1 — Supporting Information [file ADVS-11-2305183-s001.pdf]

ADVANCED SCIENCE
Open Access

Supporting Information

for *Adv. Sci.*, DOI 10.1002/advs.202305183

A H_2S-Generated Supramolecular Photosensitizer for Enhanced Photodynamic Antibacterial Infection and Relieving Inflammation

*Jia Tian, Baoxuan Huang, Lei Xia, Yucheng Zhu and Weian Zhang**

Supporting Information

A H_2S-generated Supramolecular Photosensitizer for Enhanced Photodynamic Antibacterial Infection and Relieving Inflammation

Jia Tian[#], Baoxuan Huang[#], Lei Xia, Yucheng Zhu, and Weian Zhang*

Shanghai Key Laboratory of Functional Materials Chemistry,

East China University of Science and Technology

Shanghai 200237, China

Materials

Carboxylatopillar[5]arene (**CP5**) and 5-(4-aminophenyl)-10,15,20-triphenylporphyrin (**TPP-NH$_2$**) were synthesized according to the previous literature.[1,2] Pyrrole, benzaldehyde, sodium nitrite ($NaNO_2$), tin(II) dichloride dihydrate ($SnCl_2$), 4-dimethylaminopyridine (DMAP), thiophosgene, 4-hydroxyphenylacetonitrile, *N*, *N*-dimethyl-*p*-phenylenediamine dihydrochloride (DMPD•2HCl) and ferric chloride ($FeCl_3$) were all purchased from Aladdin Reagents of China and used directly as received. L-Cysteine (Cys) was obtained from Thermo Fisher Scientific and WSP-1 was purchased from Hangzhou Diante Biotechnology Co., Ltd. Dichloromethane (DCM) and *N*, *N*-dimethylformamide (DMF) were dried over calcium hydride and distilled before use. Other reagents and solvents such as petroleum ether (PE), methanol (MeOH), dimethyl sulfoxide (DMSO) and ethyl acetate (EA) are analytical grade and used without further purification. Other chemicals were obtained from Sinopharm Chemical Reagent Co. Ltd (SRC) and used as received.

Methods

The ^{1}H nuclear magnetic resonance (^{1}H NMR) spectra were recorded using a BRUKER AV400 Spectrophotometer (400 MHz, 298K) with tetramethylsilane (TMS) as an internal reference. The ultraviolet-visible (UV-*vis*) absorption spectra were determined on a Thermo Scientific Evolution 220 spectrophotometer, and fluorescence spectra measurements were performed on a Varian's Cary Eclipse fluorescence spectrophotometer. Dynamic light scattering (DLS) and zeta potential measurements were carried out using a Beckman Coulter Delasa Nano C particle analyser and samples were characterized in an aqueous solution. All the measurements were carried out at room temperature. Transmission electron microscopy (TEM) analysis was performed on a JEOL JEM-1400 electron microscope operated at 100 kV. Scanning electron microscopy (SEM) analysis was conducted with Scanning Electron Microscope--Energy Dispersive X-ray Spectroscope (S-3400N).

Synthesis of TPP-CN

Synthesis of TPP-NCS: **TPP-NH$_2$** (150 mg, 0.24 mmol) and DMAP (12.2 mg, 0.1 mmol) were dissolved in dry CH_2Cl_2 and allowed to stir at room temperature for 20 minutes under a nitrogen atmosphere. Thiophosgene (22 μL, 0.28 mmol) was added to the solution, and the mixture was stirred at room temperature for 1 h. Then the reaction solution was washed with saturated ammonium chloride aqueous solution (for twice) and sodium chloride aqueous solution (for twice). The organic layer concentrated by rotary evaporation and purified by column chromatography to obtain **TPP-NCS**. Yield: 150 mg, 93.1%.

Synthesis of TPP-CN: 4-Hydroxyphenylacetonitrile (95 mg, 0.71 mmol) was dissolved in 5 mL of dry THF and placed in nitrogen atmosphere, then a certain amount of sodium hydride was added to the solution. After stirring for 30 min, **TPP-NCS** (95 mg, 0.14 mmol) was dissolved in 5 mL of dry THF and added into the reaction system. The deionized water was slowly added dropwise to quench the reaction after reacting at room temperature for 1 h. Subsequently, the crude product was obtained by extraction with DCM and rotary evaporation, which was further purified by column chromatography (EA: PE = 1:4). Yield: 38 mg, 33.4%.

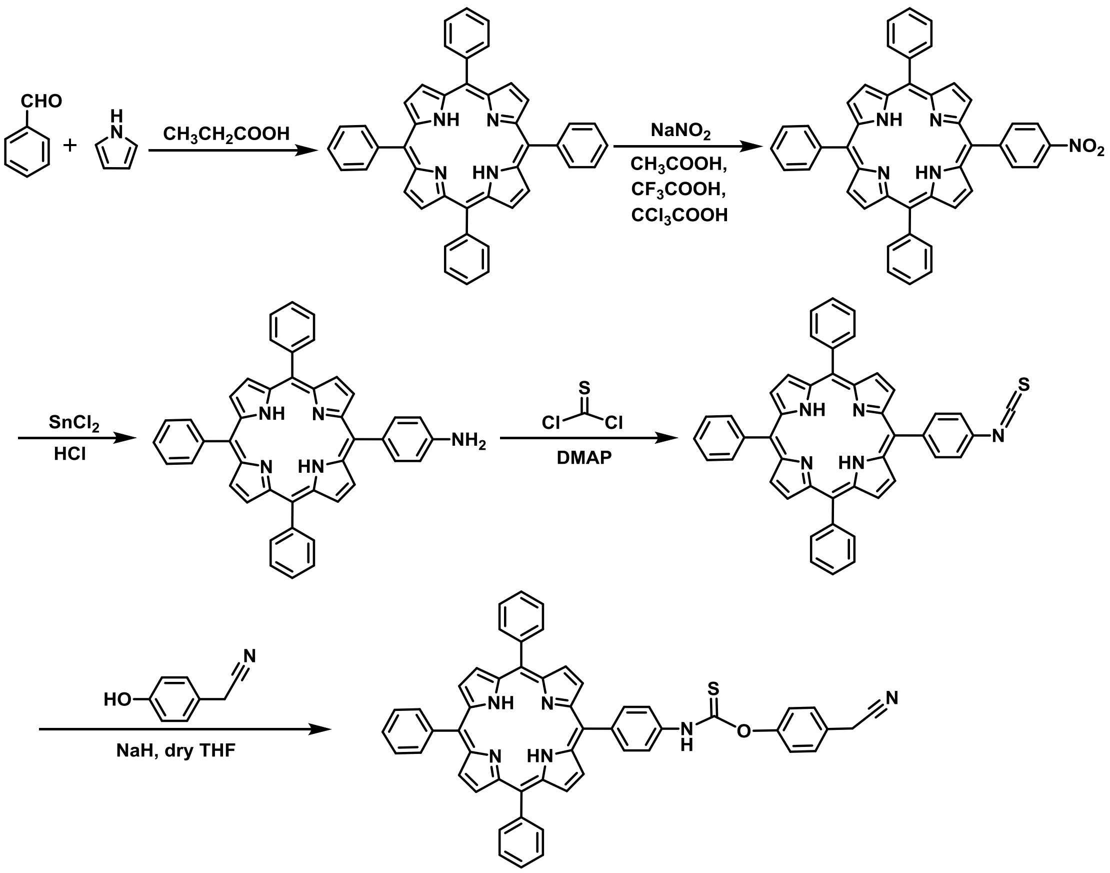

Scheme S1. Synthesis of **TPP-CN**.

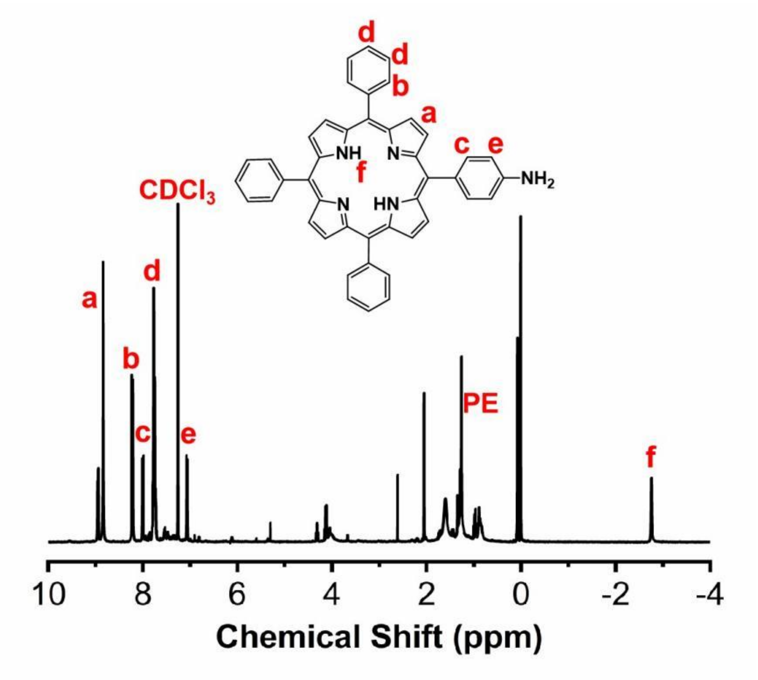

Figure S1. ^{1}H NMR of **TPP-NH$_2$**.

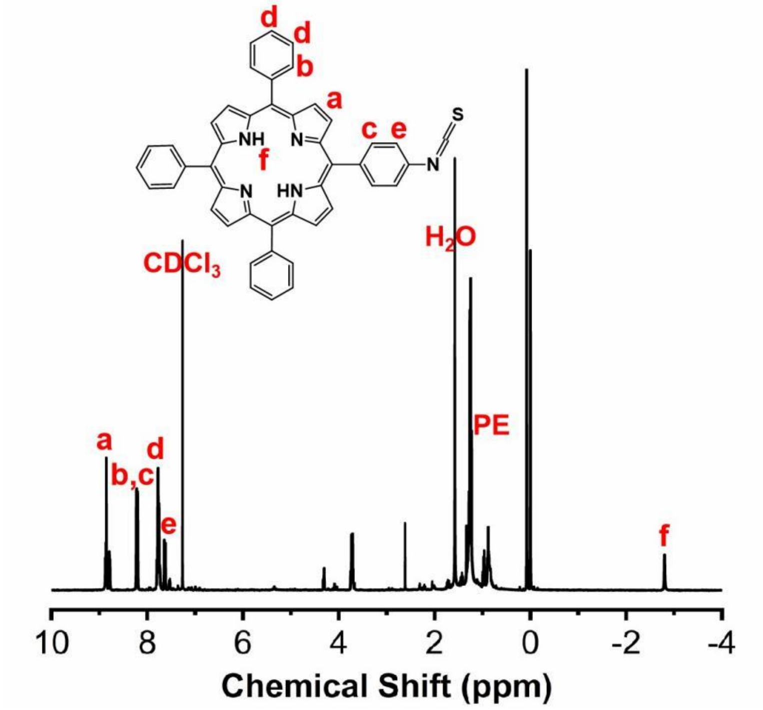

Figure S2. ^{1}H NMR of **TPP-NCS**.

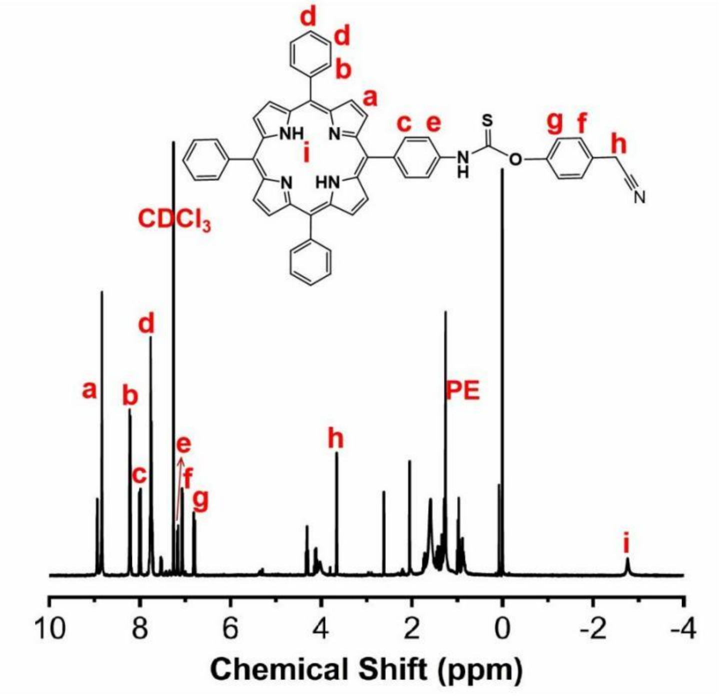

Figure S3. ^{1}H NMR of **TPP-CN**.

Investigation of the interactions between CP5 and TPP-CN

NMR titration, UV-*vis* titration and fluorescence titration experiments were utilized to study the complexation between **CP5** and **TPP-CN**.

^{1}H NMR titration: Because of the poor solubility of **TPP-CN** in D_2O, the model guest 4-hydroxyphenylacetonitrile (***G_M***) was used to investigate the host-guest complexation by ^{1}H NMR. We kept the concentration of guest fixed at 3.0 mM, and **CP5** had a varying concentration of 0, 1.0, 2.0, 3.0 and 5.0 mM. The ^{1}H NMR spectra (400 MHz, D_2O, 298 K) results were shown in **Figure 1**.

UV-vis titration: In a mixed solvent of DMF/H_2O (1/1000, v/v), the concentration of **TPP-CN** was fixed at 1×10^{-5} M, and **CP5** had a varying concentration (0, 0.25, 0.5, 0.75, 1.0, 1.5, 2.0, 2.5, 3.0 $\times10^{-5}$ M). The changes of UV-*vis* absorption were recorded on a UV-*vis* spectrometer.

Fluorescence titration: The fluorescence spectrum was performed to investigate the host-guest complexation constant (K_a) between **CP5** and **TPP-CN**. In a DMF/H_2O (1/1000, v/v) mixed solution, **TPP-CN** had a constant concentration of 1×10^{-5} M, while **CP5** had a varying concentration (0, 0.25, 0.5, 0.75, 1.0, 1.25, 1.5, 2.0, 2.5, 3.0×10^{-5} M). The complexation constant K_a was calculated by a nonlinear curve-fitting method.

Singlet oxygen generation test

1,3-Diphenylisobenzofuran (DPBF) as a singlet oxygen scavenger, was used to detect the generation of singlet oxygen ($^{1}O_2$) in the mixed solution of DMF/H_2O (1:1000, v/v) (1‰ DMF was used to improve the solubility of **TPP-CN**). The capacity of $^{1}O_2$ production was compared by monitoring the UV-*vis* absorption change at 425 nm of DPBF in the sample solution under a 660 nm laser.

The concentration of sample solution: **TPP-CN/CP5** assembly in mixed solution,

TPP-CN = 1.0 × 10^{-2} mM, **CP5** = 1.0 × 10^{-2} mM.

H_2S detection

The colorimetric methylene blue (MB) assay was used to determine the release of H_2S.[3] Briefly, 1 mL of **TPP-CN/CP5** assembly solution (20 μg/mL) was mixed with 1 mL of 1% (w/v) $Zn(OAc)_2$. MB was then formed by the addition of N,N-dimethyl-p-phenylenediamin dihydrochloride (DMPD•2HCl (20 mM)) and $FeCl_3$ (30 mM). After incubation at room temperature for 15 min, the absorbance at 665 nm was measured.

WSP-1 was used as an intracellular H_2S probe to detect bacterial intracellular H_2S. The bacterial suspension was washed with PBS for 3 times and adjusted to a concentration of 10^7 CFU mL^{-1}. After cultured with 80 μg/mL of **TPP-CN/CP5** for 4 h, Cys (100 μM) and WSP-1 (10 μM) were added, followed by incubation for 30 min. Subsequently, the obtained suspension was transferred to a confocal dish and observed by a confocal laser scanning microscope (CLSM). (Ex/Em: 465/515 nm)

Culture of bacteria

Methicillin resistant *Staphylococcus aureus* (MRSA, Gram-positive) and *Escherichia coli* (*E. coli*, Gram-negative) were cultured in liquid lysogeny broth medium (LB) and incubated on a constant temperature shaker overnight with stable shaking at 180 rpm at 37 °C. The concentration of bacteria was determined by optical density measured at 600 nm ($OD_{600\ nm}$).

Cell culture and biocompatibility test

Mouse fibroblast L929 cells were chosen as model cells. The cells were cultured in Dulbecco's modified Eagle's medium (DMEM) containing 10% fetal bovine serum (FBS) and 1% penicillin/streptomycin at 37 °C in a humidified environment containing 5% CO_2.

To assess the toxicity of **TPP-CN/CP5** nanoparticles and Cys, the L929 cells were plated into 96-well plates and incubated with different concentration of **TPP-CN/CP5**

nanoparticles and Cys solution. After 24 h of incubation with different samples, cell viability was assayed using a 3-(4,5-dimethylthiazol-2-yl)-2,5-diphenyltetrazolium bromide (MTT) reagent. Untreated cells (0 μg/mL) were used as the 100% viability control.

Biofilm dissipation experiment

MRSA was cultured with LB medium in the plate at 37 ℃ for 48 h to form the biofilm. Followed by removing the medium and rinsing unbound bacteria with PBS, the obtained MRSA biofilm was incubated with PBS or **TPP-CN/CP5** nanoparticles for 12 h. After irradiation with 660 nm light for 10 min, the biofilm was washed with PBS and the residual biofilm was evaluated by 1.0% crystal violet. Subsequently, the stained biofilm was washed 3 times with PBS and dissolved by adding 1.0 mL of ethanol, and the remaining biofilm was detected by measuring the absorbance at 590 nm. In addition, biofilm dissipation was further observed under confocal laser scanning microscopy (CLSM) by staining biofilm and bacteria with fluoresceine isothiocyanate (FITC)-labeled concanavalin A (ConA-FITC) and propidium iodide (PI), respectively.

In vitro **anti-inflammatory activity study**

The anti-inflammatory activity of supramolecular photosensitizers was evaluated by the expression level of inflammatory cytokines TNF-α and IL-6 and detected by ELISA assay. RAW 264.7 cells (a macrophage cell line) were cultured in DMEM in a humid environment at 25 ^{o}C containing 5% CO_2. Lipopolysaccharide (LPS) was selected as an inflammatory stimulator. RAW 264.7 cells were inoculated into 96-well plates at a density of 1×10^4 cells/well, cultured for 24 h, and incubated with 0.5 μg/mL LPS for 8 h, for inducing inflammation of RAW 264.7 cells to simulate acute inflammation condition. The cells pretreated with LPS were incubated with different sample solutions for 12 h. The supernatant of cell culture medium was collected and the levels of TNF-α and IL-6 in each well were tested via a commercial ELISA assay

according to the instructions. RAW 264.7 cells cultured in DMEM medium without LPS stimulation were used as a control group (Group Contr.), and the proinflammatory cytokine levels in this group were determined and normalized as "100%". Other groups were simulated by LPS. Group "0" referred to the cells only simulated by LPS without the treatment of TPP-CN/CP5 and Cys. Group "30" and "60" referred to the cells treated by LPS, Cys (100 μM), and TPP-CN/CP5 at 30 μg/mL and 60 μg/mL, respectively. Group "TPP-CN/CP5" referred to the cells simulated by LPS and treated by TPP-CN/CP5 at 60 μg/mL. And Group "Cys" referred to the cells simulated by LPS and treated by Cys (100 μM). All measurements were performed in triplicate.

Animal experiments

All animal experimental procedures were carried out in accordance with Chinese legislation on the Use and Care of Research Animals (Document No. 55, 2001), and institutional guidelines for the Care and Use of laboratory animals established by the East China University of Science and Technology Animal Studies Committee.

Female BALB/c mice (7-8 weeks old) were raised in a pathogen free environment under controlled temperature (24 °C). Then part of the dorsal hair of the mice were shaved to construct a skin wound model with diameter of about 1 cm. The wounds were treated with PBS containing MRSA (10^8 CFU) to induce bacterial infection. All mice were raised in single cages after wounding, and the day of setting model was defined as the 0 day. The mice were randomly divided into five groups (3 mice in each group) for different treatments: (1) PBS (control group); (2) **TPP-CN/CP5**; (3) **TPP-CN/CP5+L** (light); (4) **TPP-CN/CP5+Cys**; (5) **TPP-CN/CP5+L+Cys**. Among them, **TPP-CN/CP5** sample solution was 60 μg/mL (100 μL) and Cys was 100 μM. The light group was irradiated with 660 nm light for 15 min. After light irradiation, L-cysteine (100 μM) was immediately added in the TPP-CN/CP5+L+Cys group. To record the healing process of wounds, the size of wound was measured by a caliper and photographed. On the last day, the skin tissues of mice in different groups were

fixed with 10% formaldehyde and made into tissue sections for histological hematoxylin and eosin (H&E) staining analysis. In addition, the immunohistochemical staining was used to measure the pro-inflammatory cytokines within the harvested tissue by using mouse anti-TNF-α and mouse anti-IL-6.

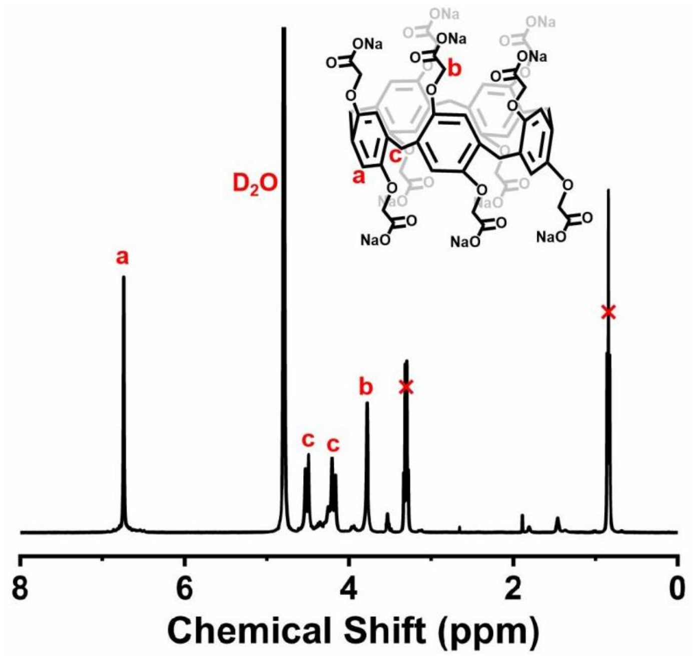

Figure S4. ^{1}H NMR of **CP5**.

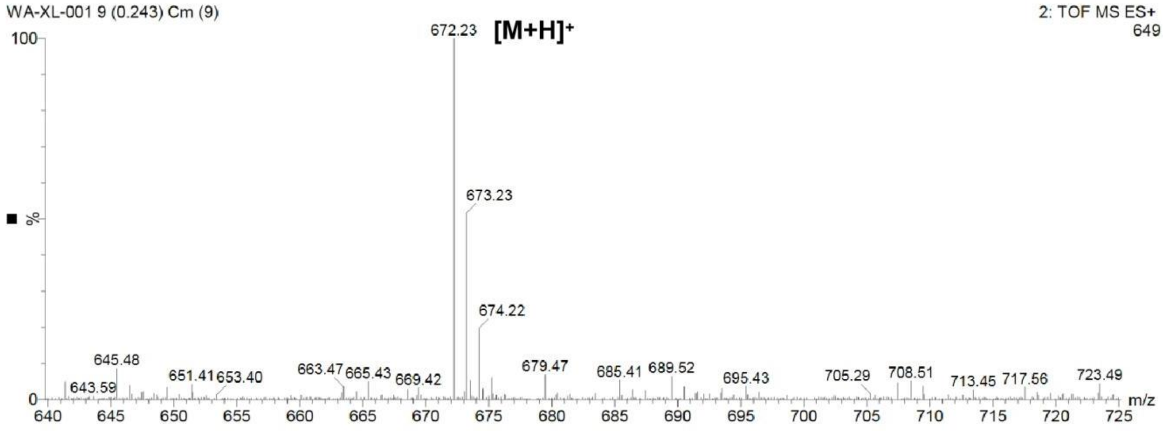

Figure S5. MALDI mass spectrum of **TPP-NCS**.

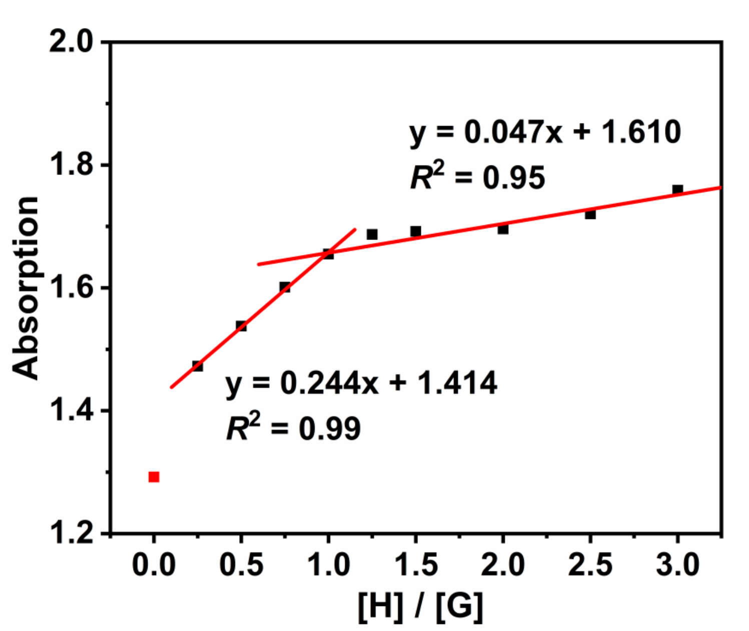

Figure S6. Job's plot showed a 1:1 stoichiometry of complexation between **CP5** and **TPP-CN** in aqueous solution (according to UV-*vis* spectrum titration).

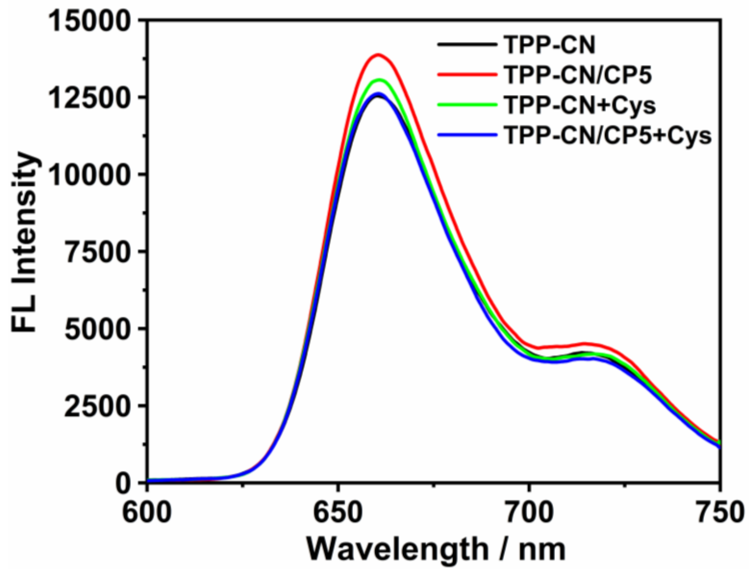

Figure S7. Fluorescence spectra of the **TPP-CN** and **TPP-CN/CP5** assemblies (dissolved in DCM) at room temperature with or without the addition of Cys.

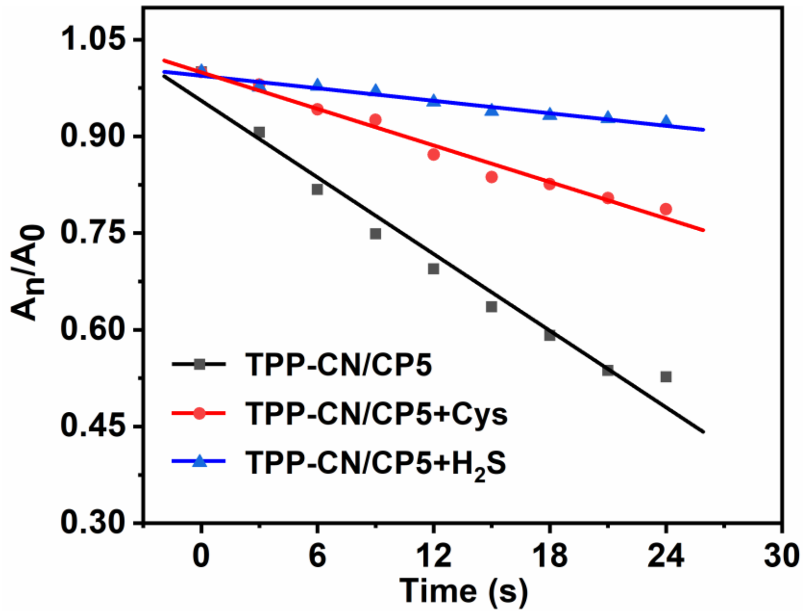

Figure S8. Degradation rates of DPBF in different solutions under light irradiation.

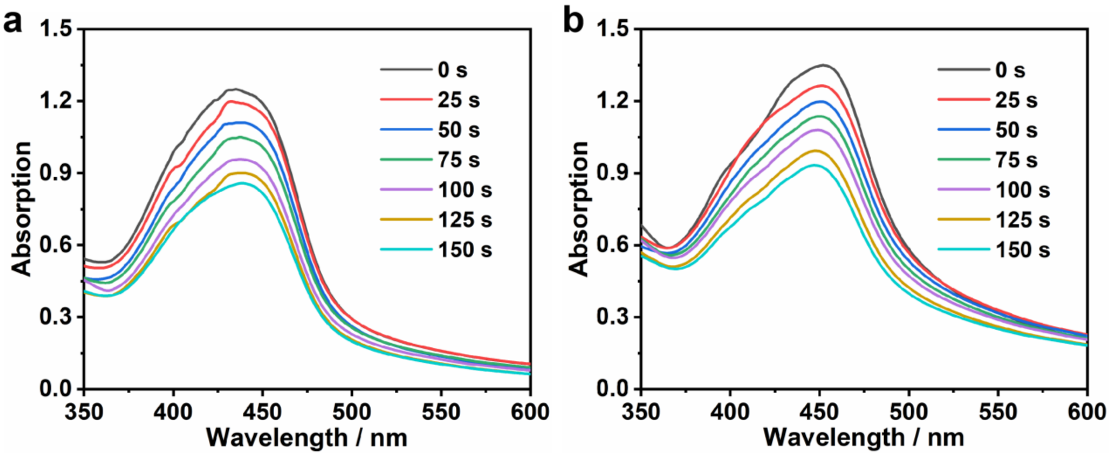

Figure S9. UV-*vis* spectrum changes of DPBF (detection of singlet oxygen production) in **(a)** TCPP(Na_4) aqueous solution and **(b)** TCPP(Na_4)+Cys solution under 660 nm light irradiation.

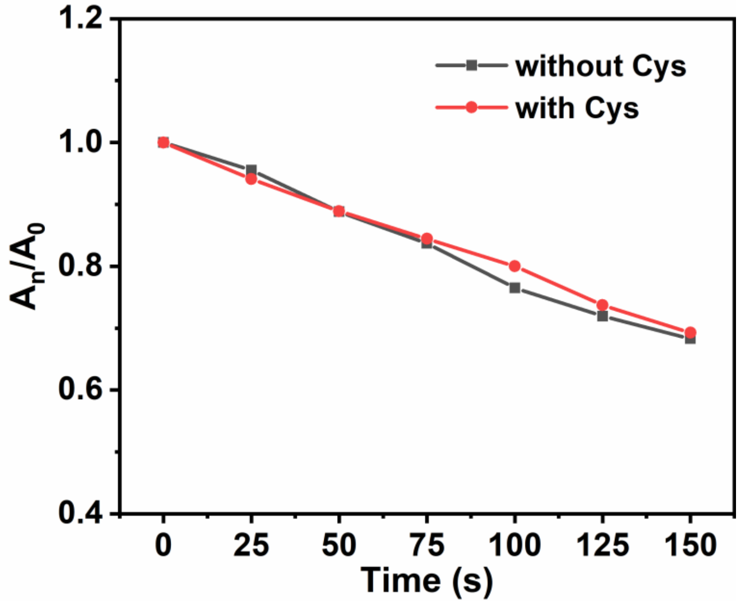

Figure S10. Degradation rates of DPBF in TCPP(Na_4) aqueous solution with or without Cys under light irradiation.

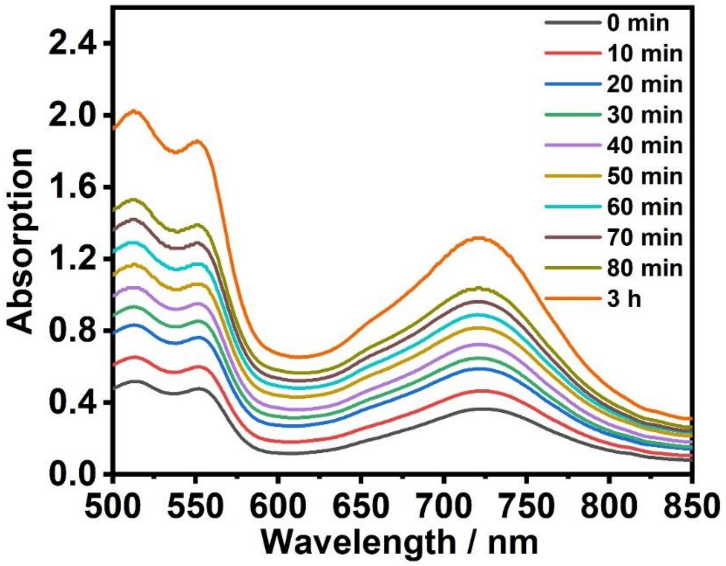

Figure S11. UV-vis absorption spectra for H_2S release detected by the MB method (TPP-CN/CP5 assemblies). In sample solution, the concentration of TPP-CN and Cys was 10 μg mL^{-1} and 100 μM, respectively.

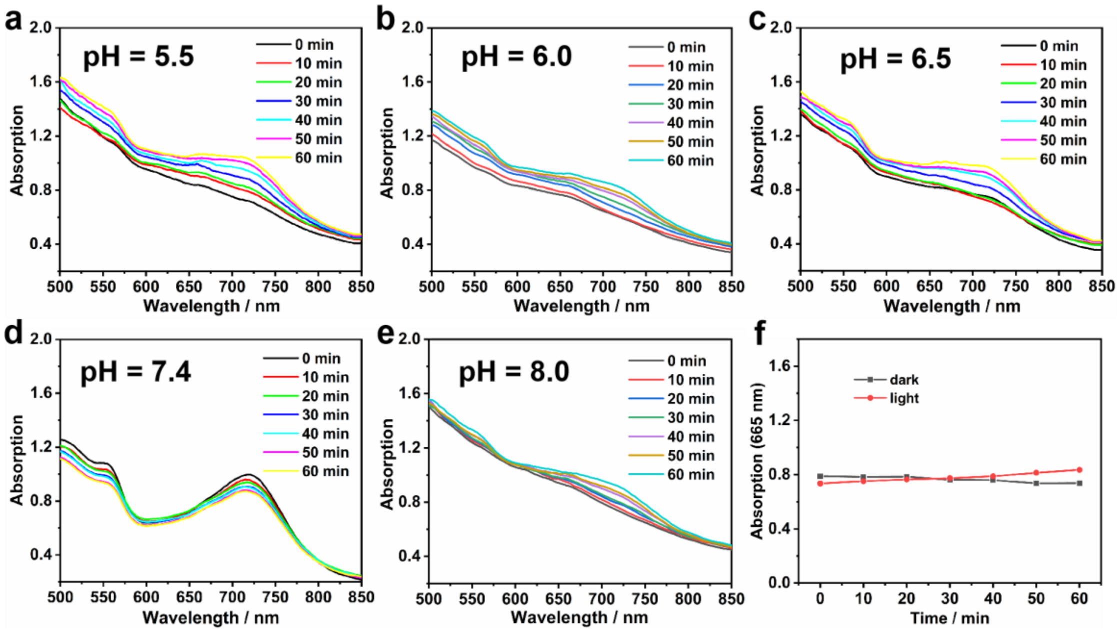

Figure S12. **a-e)** The stability of **TPP-CN** assemblies (10 μg mL^{-1}) at different pH values was evaluated by the MB method for H_2S detection. **f)** The stability of **TPP-CN** assemblies (10 μg mL^{-1}) with or without 660 nm light irradiation.

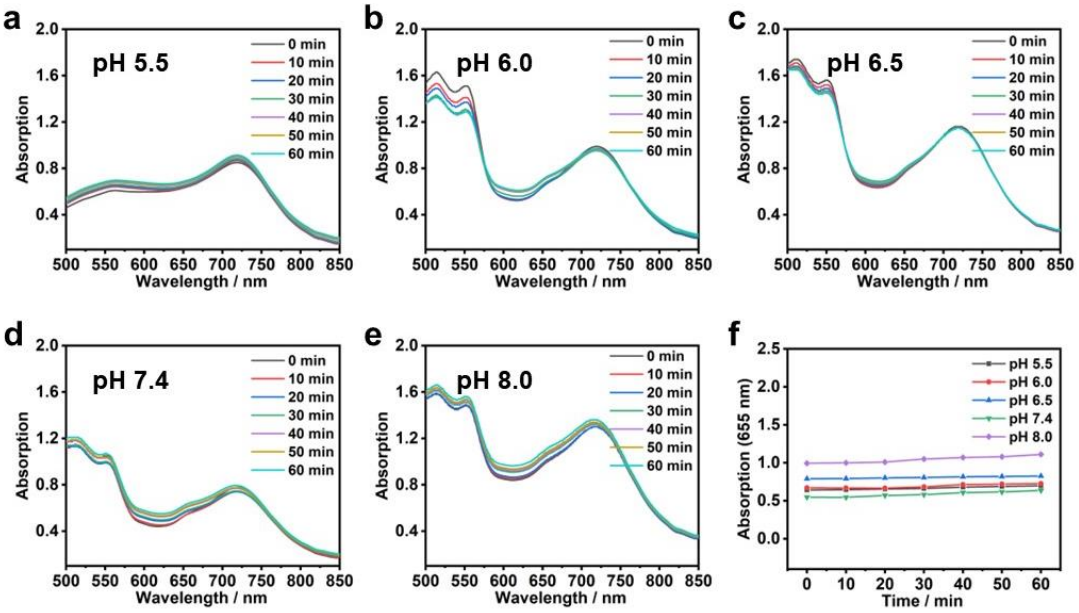

Figure S13. a-e) The stability of TPP-CN/CP5 assemblies (TPP-CN, 10 μg mL^{-1}) at different pH values was evaluated by the MB method for H_2S detection. f) Summary of the variation of absorbance at 665 nm of H_2S detection solutions (containing TPP-CN/CP5 self-assemblies) in the absence of Cys at different pH values.

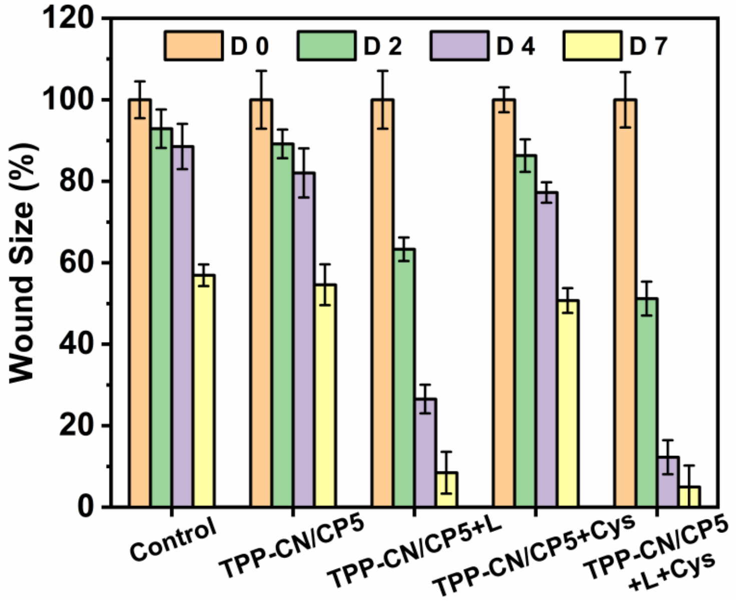

Figure S14. Changes of wound size in different groups of mice with days.

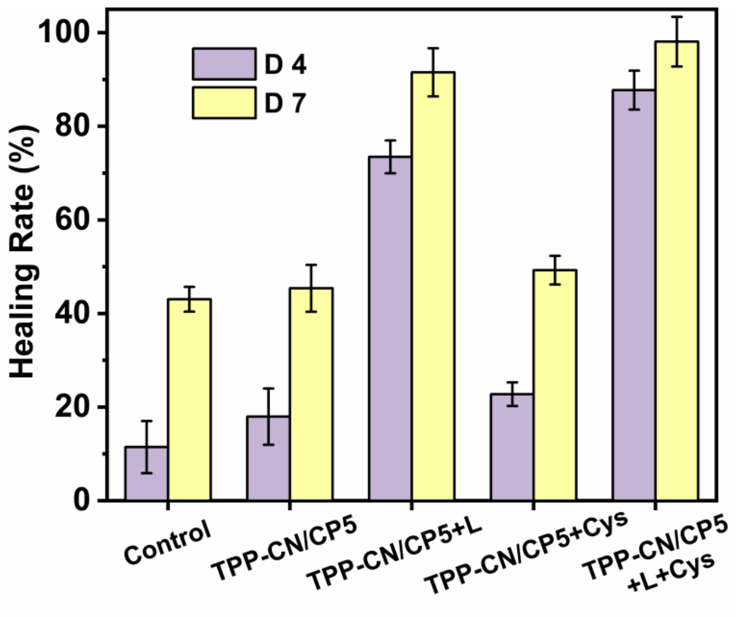

Figure S15. The healing rate of wounds in different groups of mice on the 4th and 7th day.

Reference

[1] L. Xia, J. Tian, T. Yue, H. Cao, J. Chu, H. Cai, W. Zhang, *Adv. Healthc. Mater*. **2021**, 2102015.

[2] Y. Xue, J. Tian, Z. Liu, J. Chen, M. Wu, Y. Shen, W. Zhang, *Biomacromolecules* **2019**, *20*, 2796–2808.

[3] T. He, X. Qin, C. Jiang, D. Jiang, S. Lei, J. Lin, W. G. Zhu, J. Qu, P. Huang, *Theranostics* **2020**, *10*, 2453–2462.
